# Incorporation of Eu(III) into Calcite under Recrystallization conditions

**DOI:** 10.1038/srep33137

**Published:** 2016-09-13

**Authors:** S. E. Hellebrandt, S. Hofmann, N. Jordan, A. Barkleit, M. Schmidt

**Affiliations:** 1 Helmholtz-Zentrum Dresden - Rossendorf, Institute of Resource Ecology, Bautzner Landstraße 400, 01328 Dresden, Germany

## Abstract

The interaction of calcite with trivalent europium under recrystallization conditions was studied on the molecular level using site-selective time-resolved laser fluorescence spectroscopy (TRLFS). We conducted batch studies with a reaction time from seven days up to three years with three calcite powders, which differed in their specific surface area, recrystallization rates and impurities content. With increase of the recrystallization rate incorporation of Eu^3+^ occurs faster and its speciation comes to be dominated by one species with its excitation maximum at 578.8 nm, so far not identified during previous investigations of this process under growth and phase transformation conditions. A long lifetime of 3750 μs demonstrates complete loss of hydration, consequently Eu must have been incorporated into the bulk crystal. The results show a strong dependence of the incorporation kinetics on the recrystallization rate of the different calcites. Furthermore the investigation of the effect of different background electrolytes (NaCl and KCl) demonstrate that the incorporation process under recrystallization conditions strongly depends on the availability of Na^+^. These findings emphasize the different retention potential of calcite as a primary and secondary mineral e.g. in a nuclear waste disposal site.

The long-term safety of geological nuclear waste disposals has to rely on a detailed and thorough understanding of the processes governing the migration of radionuclides through the geo/biosphere. These reactions can include adsorption and surface precipitation as well as incorporation within the bulk of a material. Calcite, one of earth’s most abundant minerals, has long been known for its potential to incorporate other cations than calcium^1^,^2^,^3^ and is present in significant amounts in clay rock formations^4^ as well as as a degradation products of cementitious materials^5^. Consequently, calcite may play a significant role as a retention barrier for the transport of contaminants in the environment. Its performance as such a barrier will depend on the specific conditions of the interaction. Calcite is also of high relevance for many technical processes, where it occurs as scales, which have been shown to form solid solutions with certain contaminants, e.g. radium and other naturally occurring radioactive materials^6^,^7^. In many geological and geotechnical scenarios, such as nuclear waste storage in deep geological formations, the contaminants will interact with pre-existing calcite close to thermodynamic equilibrium with its contacting solution, under conditions where the incorporation process can be expected to be controlled by the mineral’s recrystallization rate.

Plutonium and the minor actinides neptunium, americium, and curium are expected to contribute most significantly to the long-term radiotoxicity according to performance safety assessments^8^,^9^. Under the reducing conditions expected for a deep geological disposal site the preferred oxidation state for Am and Cm, but also possibly for Pu is +3. Europium has often been chosen as a homologue for trivalent actinides because of its similar ionic charge and radius^10^,^11^, as well as for its remarkable luminescent properties^12^. The capacity of calcite to incorporate guest ions with similar ionic radius compared to Ca^2+^ (e.g. Eu^3+^, Am^3+^, and Cm^3+^) under growth, phase transformation and even dissolution conditions was already evidenced in the past^1^,^3^,^13^,^14^,^15^,^16^,^17^,^18^,^19^,^20^,^21^,^22^,^23^,^24^,^25^. These processes represent an important retention mechanism for many contaminants, such as Pb^2+^ or Cd^2+^, but also trivalent actinides (Pu^3+^, Am^3+^, Cm^3+^) relevant to long term nuclear waste storage. In these previous studies it was shown that trivalent rare earth elements and actinides can be taken up by the calcite host lattice structurally. Natural mineral specimen contain considerable amounts of rare earths depending strongly on their origin. These findings imply that under natural conditions, mechanisms must be at work by which dissolved metal ions in contact with calcite at equilibrium incorporate into the material over time.

Moreover, the dissolution and precipitation behavior of abundant minerals like calcite under different fluid compositions is important for the evaluation of geothermal energy production sites^26^,^27^. Also for the long-term storage of atmospheric CO_2_ in geological formations calcite and other carbonates with their slow kinetics of mineral-fluid reactions play a significant role^28^.

The interaction of Eu^3+^ as well as Cm^3+^ with calcite synthesized in mixed flow reactors^3^,^13^,^29^ or formed by mineral phase transition^14^ has previously been investigated at the molecular level by site-selective time-resolved laser-induced fluorescence spectroscopy (TRLFS). Here, three main species were identified. Species A corresponds to a surface species with ~2 water molecules left in its hydration shell and for Eu showed a peak maximum for the ^7^F_0_ → ^5^D_0_ transition at 578.1 nm. Species B (λ_exc_(Eu) = 578.4 nm) was found to be incorporated within the strongly distorted calcite structure. Finally, species C (λ_exc_(Eu) = 579.6 nm) was found to be located on the Ca^2+^ site on the calcite lattice, with an almost undisturbed octahedral symmetry, identifiable by its characteristic splitting pattern. For both species B and C, Na^+^ was required for charge compensation during the incorporation, suggesting a mechanism where two Ca^2+^ ions were replaced by one Eu^3+^ atom and one Na^+^ atom. Atomistic simulations by Vinograd *et al*.^30^ find a dolomite-like layered structure for the hypothetical NaEu(CO_3_)_2_ end member, and they suggest the formation of such domains in the solid solution. The Ca^2+^ lattice site in dolomite has a D_3d_ point symmetry, as opposed to the C_3i_ symmetry of the same lattice site in calcite. Therefore, Eu in such a crystallographic environment should exhibit a splitting pattern similar to that of species C in calcite.

Where calcite occurs as a primary phase, interaction will occur close to equilibrium. Studies dedicated to the interaction of Eu^3+^ or Cm^3+^ with calcite under recrystallization conditions are scarce. Our previous work^29^ studied the sorption of Eu^3+^ on a single calcite powder in 0.01 mol L^−1^ NaClO_4_ at different contact times, i.e. one day, two weeks and one month at recrystallization conditions. In the excitation spectra, a broad peak centered at 579.3 nm with at least two species exhibiting partially preserved hydration shells were observed. Interaction was found to be dominated by a continuum of chemically similar adsorbed species as well as beginning incorporation into the bulk structure. An impact of the reaction time on the number of species and their lifetimes was noticed, but the transition was too slow to be probed meaningfully in the allotted time.

Earlier Stumpf and Fanghänel^15^ described a similar process of Cm^3+^ interaction with calcite under recrystallization conditions. Via TRLFS of curium’s ^6^D_7/2_ → ^8^S_7/2_ transition they observed a sorption species with two and an incorporation species with no water molecules bound to the Cm^3+^, respectively. Over a contact time of 6 months the incorporation of Cm^3+^ in to the crystal bulk progressed at the cost of the sorption species. The incorporation species identified by Stumpf and Fanghänel^15^ has its emission maximum at 618.0 nm, clearly distinct from the two incorporation species identified in co-precipitation experiments, species B at 616.3 nm and species C at 624.3 nm.

Piriou *et al*.^31^ also investigated the interaction of Eu^3+^ with calcite. Batch sorption experiments were conducted at 50 °C and samples subsequently analyzed by site-selective TRLFS. Eu^3+^ was found to be incorporated into a hydrated and/or hydroxylated surface layer, with two distinct site families. Substitution of Eu^3+^ for Ca^2+^ in the lattice framework was also in evidence. Nevertheless, sites A and B revealed by Schmidt *et al*.^3^ and Marques Fernandes *et al*.^13^ under growth conditions were not observed by Piriou *et al*.^31^ Consequently, there are already clear hints in the literature highlighting the trend that speciation and interaction of Eu^3+^ and Cm^3+^ with calcite evidently is strongly affected by experimental conditions.

To further investigate the impact of surface reactivity and recrystallization kinetics of calcite on the incorporation of Eu^3+^ three calcite powders differing in their specific surface area (SSA) and impurities content were chosen and their reactions at near-equilibrium conditions studied. The evolution of the speciation was followed as a function of reaction time, starting from one week up to two months, and for some samples up to three years. In order to probe the relevance of the coupled substitution mechanism identified by Schmidt *et al*.^3^, some experiments were additionally conducted using KCl instead of NaCl as background electrolyte.

## Results

### Characterization of the solid phases

Three calcite powders (C1, C2, and C3) were chosen for this study. C1 and C3 were obtained as powders from Merck, Germany and Solvay, Germany, respectively. C2 was obtained from Ward’s Science, USA as a single crystal and ground to a grain size < 63 μm. All solids were characterized by powder XRD, BET, and SEM. In addition the impurity inventory was characterized by ICP-MS after dissolution of the minerals (for more details see Table S1 in the supplementary information (SI)). The sample characteristics are summarized in Table 1.

**Figure 1:**
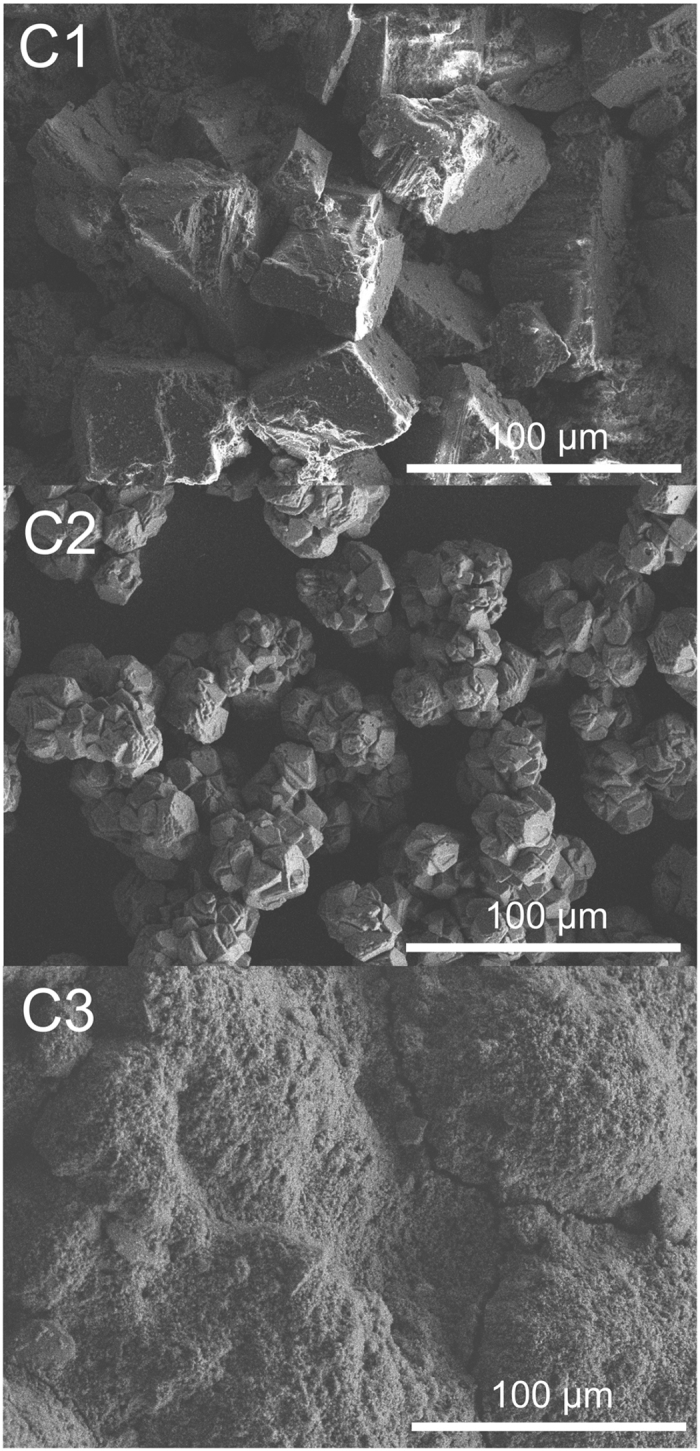
Scanning electron micrographs of the three calcite powders C1, C2, and C3. The typical rhombohedral shape of calcite is clearly visible in Fig. 1(a) with the largest grains being approximately 100 μm in size.

The powder X-ray diffraction patterns of C1, C2, and C3 showed the presence of well-crystallized calcite, with no trace of other CaCO_3_ allotropes such as aragonite or vaterite (for more details see Figure S1 in SI). The specific surface area (SSA) of the powders was found to be 0.6 ± 0.1, 1.1 ± 0.1 and 18.0 ± 0.1 m^2^ g^−1^ for calcite C1, C2, and C3, respectively. The total organic content of calcites C1 and C2 were lower than 0.1 mg g^−1^, while that of calcite C3 was 0.13 ± 0.04 mg g^−1^. Representative micrographs showing the decreasing crystallite size from C1 to C3 are presented in Fig. 1.

Sample C1 exhibited the overall lowest amount of impurities, the highest being Mg, Sr and Fe (7.5 to 14.5 μg g^−1^). Concerning C2, impurities in Na, Mg, Si, K, Sr, Cs, Fe and Zn (10.4 to 142 μg g^−1^) were present. In addition to the aforementioned elements for C2, material C3 had also minor impurities in Ba and Mn. The highest concentrations were found for Na (109 μg g^−1^), Mg (1390 μg g^−1^) and Si (389 μg g^−1^).

### Calcite recrystallization rates

**Figure 2:**
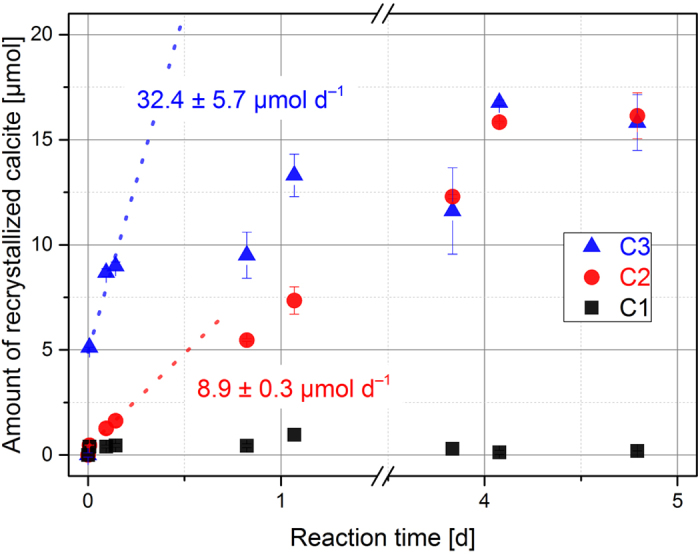
Amount of recrystallized calcite (μmol) as a function of time (■) C1, (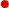) C2, and (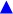) C3 (m/v = 2.5 g L^−1^, 0.01 mol L^−1^ NaCl).

The exchange rate of calcium ions between the solid and liquid phase was determined by tracer experiments using the beta emitter ^45^Ca. It is commonly assumed that the kinetics of this exchange is directly linked to the recrystallization rate and therefore the reactivity of calcite. The amount of recrystallized calcite material calculated from ^45^Ca activity in solution as a function of reaction time is presented in Fig. 2.

As also seen in the study by Berner *et al*.^32^, three stages of recrystallization kinetics can be identified. The highest rate is observed within the first hours of the reaction, followed by a decrease of the rate and eventually equilibrium which is reached when the concentration of the radioactive tracer in solution remains constant. The first two phases of this reaction are caused by the different availability of tracer ions in solution and solid. First, an excess supply of ^45^Ca in solution leads to more incorporation than dissolution. With time, significant amounts of the tracer have become incorporated into the bulk and are then subject to dissolution and resolvation decreasing the overall recrystallization rate. Once chemical equilibrium is reached between dissolution and incorporation of ^45^Ca, i.e. the same molar amount of ions is incorporated as is dissolved, no more information can be drawn from the data. When the second phase of the process (1.7–25.7 h) is used for data fitting, both calcites C2 and C3 exhibit significantly higher rates compared to C1. Material C1 recrystallizes much slower and equilibrium is not reached within the reaction time. Clearly, C1’s recrystallization rate is significantly slower compared to the other materials, and we would expect this material to show the slowest surface reaction kinetics. Rate determination here is only slightly above detection limit. The rates of calcites C2 and C3 are rather high and equilibrium is reached rapidly. The specific rates, however, differ from each other with C3 being the most reactive.

### Species identification and reaction kinetics

**Figure 3:**
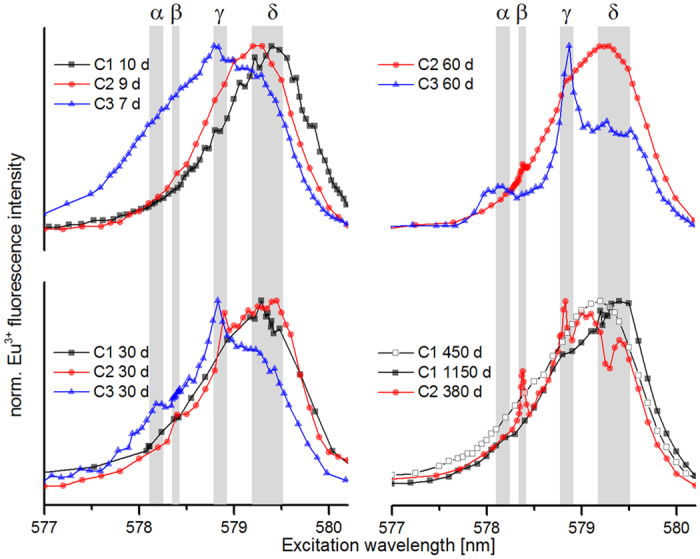
^7^F_0_→^5^D_0_ Excitation spectra after 7 days (C1-C3), 30 days (C1-C3), 60 days (C2, and C3) and after longer reaction times (C1 450 and 1150 days; C2 380 days).

The 3 different calcites (C1, C2, and C3) sorbed nearly all Eu^3+^ of the solution independent of the reaction time (see Table S3 in SI). The excitation spectra of the (^5^D_0_ → ^7^F_0_) transition for all samples recorded at various reaction times are presented in Fig. 3. In total, four species named α–δ were identified. Their occurrence and relative distribution depends strongly on the material used, and hence the recrystallization rate. The characteristics of these species are summarized in Table 2.

#### Seven to ten days

Already after ca. one week the spectra show significant differences between C1, C2, and C3, while all spectra show a broad distribution. For the calcite C1 with the lowest recrystallization rate, only one broad peak (Full Width at Half Maximum (FWHM) ~1 nm) is found at ~579.4 nm, which was assigned to species δ. The spectrum of sample C2 is dominated by the same species δ, but is slightly shifted to lower excitation wavelengths. The sample with the highest recrystallization rate C3, however, shows a significantly broader peak (FWHM ~ 1.6 nm), as well as a more strongly blue-shifted spectrum. A weak peak at 578.8 nm is assigned to species γ. The shoulder with higher excitation wavelength shows the presence of species δ, and the intensity at lower excitation wavelength points to one or more species present, which are not clearly resolved here. A general trend of more strongly blue-shifted spectra for the calcites with higher recrystallization rate is visible.

#### One month

After one month the different progress in the reactions becomes more apparent. For C1 the excitation spectrum shows only minor changes. A slight shoulder is now visible at lower excitation wavelength which may indicate the presence of small quantities of species γ, but the speciation remains dominated by species δ. The same applies to C2, but weak peaks indicate contributions by species γ and species β, visible at 578.4 nm. The spectrum of C3 shows more clearly resolved peaks now, dominated by species γ. Both, the shoulder at the spectral range of species δ, and the shoulder at lower excitation wavelength decrease. At 578.2 nm a small peak becomes visible, which is attributed to a fourth species α.

#### Two months

Due to the slow progress in the reaction of C1 from one week to one month, only C2 and C3 were probed after two months. The excitation spectra exhibit significant differences between C2 and C3. The excitation spectrum of C2 again shows a broad distribution with the maximum at species δ. Species γ is present, but because of the stronger blueshift of the whole spectrum species γ is not predominant. In addition, species β is recognizable as a small, but well-defined peak.

The different reaction kinetics comes out clearly at this point. The excitation spectrum of C3 now shows well defined peaks for species α and γ, as well as a shoulder corresponding to species δ. The shoulder is decreased in intensity compared to one month earlier and appears to be divided into two peaks.

#### Long-term experiments

Additional long-term experiments were conducted with C1 and C2, after 450 d and 1150 d, and 380 d, respectively to probe their slower reaction kinetics. The excitation spectrum of C1 is still broad and essentially featureless after 1.5 years (450 d), but starts to exhibit more clearly resolved features after ~3 years (1150 d). The speciation is still dominated by species δ, but the shoulder corresponding to species γ is now clearly visible. Also a small shoulder may be present, which would indicate the formation of species α. As expected the reaction proceeds quicker for C2. After one year (380 d) the excitation spectrum of C2 shows four distinguishable peaks. The dominating species is now γ, similar to C3 at the previous time steps. Species β is characterized by a sharp, narrow peak, while species δ shows a differentiation into two features similar to the same species observed for C3 after 2 months.

### Species characterization

**Figure 4:**
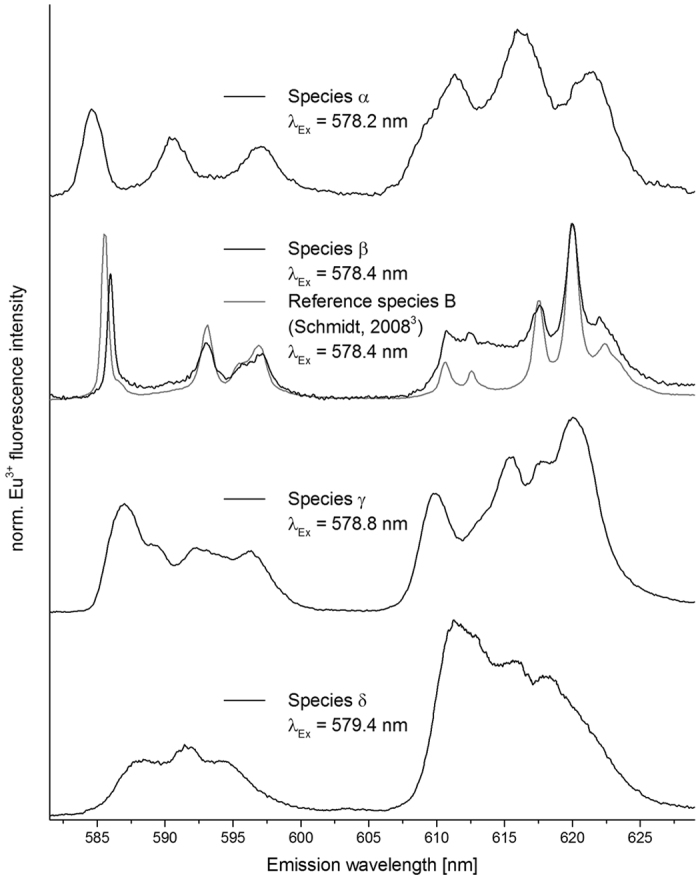
Representative emission spectra of species α–δ after selective excitation of the ^7^F_0_→^5^D_0_ transition.

Four unique species were identified throughout the reaction, which can be characterized by their emission spectra and fluorescence lifetimes. The emission spectra after direct excitation of the four different species are shown in Fig. 4, fluorescence lifetimes and additional characteristics are given in Table 2 (for fluorescence decay profiles see Figure S2 in SI).

Species α was identified in C3 after 30 and 60 days, and may also be present in C1 after ~3 years. The ^7^F_1_ band shows a 3-fold splitting indicating a low symmetry^12^, despite the fact, that the ^7^F_2_ band exhibits only a 3-fold splitting. Ligand field considerations dictate that a full splitting of the ^7^F_1_ band must be accompanied by a full splitting (5-fold) of the ^7^F_2_ band, which is likely not sufficiently resolved in our spectra, as the peaks are noticeably broadened. The long lifetime of 3700 ± 350 μs corresponds to the complete loss of the hydration of Eu^3+^ 33 and therefore with an incorporation of Eu^3+^ into the calcite crystal bulk.

Species β has its excitation maximum at 578.4 nm and appears only in material C2 as a shoulder after one and two month and as a sharp and narrow peak after more than one year of reaction time. The corresponding emission spectrum reveals a 3-fold splitting in the ^7^F_1_ band and a 5-fold split ^7^F_2_ band, this maximum splitting pattern implies a low symmetry of the ligand field surrounding Eu^3+^. The lifetime of 2450 ± 150 μs here also points at complete loss of hydration in the first coordination sphere of Eu^3+^ and therefore an incorporation into the crystal bulk. Comparison of emission spectrum and lifetime clearly identifies β as *species B*, which had been previously identified in coprecipitation experiments^3^,^29^.

Species γ is unique to the incorporation process close to equilibrium, and was identified in all 3 calcite materials. Its excitation maximum was found at 578.8 nm. To interpret its emission spectrum we have to consider an apparent overlap with species δ. This results from the broadness of the excitation peak of species δ and the small distance between the two excitation maxima. While no single species emission spectrum could be obtained, it appears a 3-fold ^7^F_1_ and a 5-fold ^7^F_2_ band can be determined, so site symmetry of species γ is low as well. Due to the inherent overlap of the emission spectra, additional interpretation of the observed splitting pattern is however not expedient. The same problem occurs in determination of the lifetimes of both species. We determine the respective lifetimes, where either species is most dominant, and we can be most confident in the determined value, but overlap remains an issue (see also discussion of lifetimes of species δ). Despite these concerns, a lifetime of 3750 ± 450 μs clearly shows a water free coordination shell of Eu^3+^, and hence bulk incorporation.

Species δ also occurs in all three calcites and dominates speciation of all samples early on, before being successively replaced by species γ, as well as α or β. The excitation maximum is always broad within the range of 579.2–579.5 nm. In the last spectrum of C2 (380 d) and C3 (60 d), respectively, the excitation peak is divided into two distinct peaks, but neither the emission spectra nor the lifetimes exhibit significant differences, upon excitation at either of the two maxima. The full splitting of ^7^F_1_ and ^7^F_2_ indicates a low symmetry.

As mentioned above determination of the lifetime of δ is difficult, due to its broad excitation range, and thus overlap with γ, and therefore is most reliable for early samples. Here we find lifetimes of ~700 ± 50 μs, corresponding to ~0.9 ± 0.5 H_2_O in Eu’s first coordination sphere. Species δ therefore can be identified as an Eu inner-sphere sorption complex on the calcite surface, the expected starting point of the metal/mineral interaction. However, with increased reaction time we determine continuously longer lifetimes up to a value of ~ 2300 ± 150 μs, which would imply no water molecules surrounding Eu^3+^, with no apparent changes in the emission spectra. It seems unlikely that an incorporation species would form so rapidly, and with such predominance even in the otherwise slowly reacting calcites C1 and C2. Therefore, we suggest the long lifetime occurs for different reasons. First of all, it is likely that strong overlap with the long-lived species γ contributes significantly to an apparently longer lifetime of δ, especially as γ becomes more dominant with longer reaction times. It is also possible that with the advancing recrystallization reaction more CO_3_^2−^ becomes available in the near-surface solution, which could potentially substitute the coordinating water molecules to form a ternary surface complex as an intermediate step to full structural incorporation. Such a substitution could also potentially cause the observed splitting of species δ’s broad excitation peak into two narrower (yet still strongly overlapping) peaks. Despite the uncertainties regarding species δ, it is evident that this species is not identical to the incorporation species C on the symmetrical Ca^2+^ lattice site^3^, which had been previously identified in our co-precipitation experiments with a similar excitation wavelength (λ_exc_ (C) = 579.6 nm).

### Charge compensation mechanism

**Figure 5:**
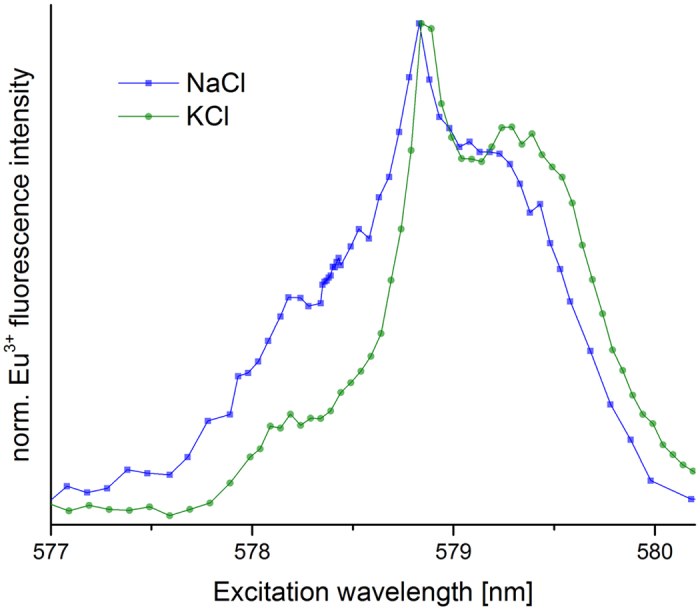
^7^F_0_→^5^D_0_ excitation spectra after 30 days reaction time of C3 with NaCl and KCl as background electrolyte. Spectra normalized to the respective peak intensity.

Incorporation species γ had not been previously identified in our studies under growth conditions. However, those studies had shown that structural incorporation of Eu^3+^ into calcite requires co-substitution with Na^+^ for charge compensation. With the fastest reacting calcite C3 we conducted a recrystallization experiment in which we replace NaCl by KCl as background electrolyte to test whether the same charge compensation mechanism plays a role under recrystallization conditions. The excitation spectra of C3 reacted with both electrolytes are displayed in Fig. 5.

The speciation of both systems is dominated by species γ, but the spectrum of the NaCl system is strongly blue-shifted compared to the KCl system. As the most strongly red-shifted species δ was identified as a sorption species, and all three more blue-shifted species (α–γ) are incorporated into the bulk, this indicates enhanced incorporation in the NaCl system relative to the KCl system. This may indicate that also under recrystallization conditions coupled substitution of Eu^3+^ and Na^+^ for two Ca^2+^ is required for incorporation. Nevertheless, incorporation remains the most significant interaction mechanism in the KCl system, likely due to a considerable amount of naturally occurring Na in calcite C3 (for impurity contents see Table S1 in SI). Also results of ICP-MS measurements show similar Eu content in both systems, NaCl and KCl (see Table S4 in SI). The suggestion of Vinograd *et al*.^30^, that a dolomite like structure forms through coupled substitution cannot be verified here, as no species with a point symmetry of D_3d_ was found in this study.

## Discussion

Our study demonstrates that interaction of Eu and calcite will result in significantly different speciation depending on whether the interaction occurs under growth conditions, or *via* recrystallization. Moreover, in the latter case speciation will also be impacted by the recrystallization rate, with a clear trend towards increased incorporation at faster recrystallization.

The initial step of the reaction is a Eu^3+^ inner sphere sorption species (species δ) on the calcite surface. Eu^3+^ is initially still in contact with water, which presumably is substituted by CO_3_^2−^ in a ternary surface complex, before Eu is buried under more crystal layers to become structurally incorporated.

The main product of the incorporation reaction is none of the species A, B, or C that were previously identified after co-precipitation of calcite in the presence of Eu^3^, but instead the new species γ was identified and characterized. The site occupied by species γ is of low symmetry and bears little resemblance to the trigonal Ca site. The newly identified incorporation mechanism must then result either in a significantly stronger distortion induced in the host crystal’s lattice, or Eu occupies a non-crystallographic site. We can exclude that the newly identified species is incorporated on a crystallographic site of a NaEu(CO_3_)_2_-dolomite domain, based on symmetry considerations.

One could speculate that the structural control of a local disturbance in the lattice is stronger in the slow process close to equilibrium, than in the faster reaction upon mineral growth. For example, Eu’s preference for specific sorption sites on the calcite surface could direct its final location in the lattice. During growth of calcite preferred sorption of cations would be expected to occur in crystallographic locations (otherwise an amorphous material would form), while in the case of recrystallization sorption may be more likely at e.g. kink sites, step edges, or other similar surface defects. Thus, Eu would be found in a distorted or non-crystallographic site from the initial sorption step onwards.

We observed two other low symmetry incorporation species, species α (C3) and species β (in C2). Species β has been observed in our previous co-precipitation experiments (as species B), but is less defined here than in the co-precipitation experiments. Species α was hitherto unknown and is always a minor species where it occurs at all. Species β only occurs for sample C2, and only after more than one year of reaction. Here, its corresponding peak is relatively strong and well defined, however. It remains unclear what the mechanism of formation of these minor species is, and whether they are transitory species, or will remain stable. At least species β only occurs in significant amounts after major contributions of γ have been found, and γ is not significantly reduced where it occurs, so evidently the two species form independently.

The effect of coupled substitution (Eu^3+^ + Na^+^ ↔ 2 Ca^2+^) has been described before^3^ and is to some extent also observed for our incorporation species. Our results comparing NaCl and KCl as background electrolyte show a strong blue shift (i.e. towards incorporation species) in the excitation spectra of the NaCl system in comparison to the KCl system. C3, the calcite used for this experiment, contains a considerable amount of Na, so coupled substitution can still proceed here as well, but to a reduced extent.

It is apparent that the formed incorporation species γ is reproducible throughout our experimental series, though the kinetics of its formation vary significantly. The higher the recrystallization rate, the faster the process of incorporation proceeds, and the more dominant species γ appears. Based on ICP-MS analysis of C1 − 3 it is also possible that the increased concentration of impurities plays a role in the incorporation process. Yet, it appears counter-intuitive to assume a high content of impurities would directly increase the potential for uptake of other contaminants by calcite. There may well be an indirect effect of the impurities though, either by providing a charge compensation pathway, or by increasing the recrystallization rate, in agreement with the overproportional release of Na from C3 in a dissolution experiment (see Table S2 in SI).

## Conclusion

Our findings conclusively show that Eu^3+^ is incorporated into calcite under recrystallization conditions. The observed speciation is different from that found in co-precipitation experiments and will depend on calcite’s recrystallization rate or reaction kinetics. After sufficiently long time speciation is dominated by species γ, independent of the time required to reach this point. Species γ is incorporated, but not on a crystallographic site. It is likely that the high content of rare earth elements, which is found in natural calcites is present predominantly in form of species γ, where incorporation occurred close to equilibrium. In addition, this means that calcite will act differently as a retention barrier, e.g. in a nuclear waste disposal site, depending on whether it is present as a component of the host rock formation and thus close to thermodynamic equilibrium, or formed later on as a secondary phase.

While a driving force for incorporation clearly exists under the recrystallization conditions studied here, it remains unclear whether species γ is more or less stable than species C, which is described as incorporation on the Ca lattice site. A conclusive answer to this question will likely have to rely on theoretical calculations.

The charge compensation mechanism appears to be unaffected by the type of incorporation species formed. As previously found for species B and C, species γ as well is influenced by the background electrolyte. This suggests, that even though we cannot assign a crystallographic site to this species, its formation must involve substitution of Ca^2+^ by Eu^3+^.

## Experimental

### Characterization of the solid phases

Calcite from Merck (calcite C1), natural calcite single crystals purchased from Ward’s Science USA (calcite C2) and industrial calcite from Solvay (calcite C3) were used in this study. Materials C1 and C3 were used as delivered, while C2 was ground and sieved to grain size < 63 μm with a ceramic mortar. The three calcites were characterized by powder X-ray diffraction (XRD) (see Figure S1 in SI). The specific surface areas (SSA) were determined by applying the Brunauer–Emmett–Teller (BET) equation with nitrogen adsorption isotherms at 77 K (Multi-point Beckman Coulter surface analyzer SA 3100). The total organic carbon content was determined by a multi N/C 2100 (Analytik Jena AG). To characterize the surface morphology of the calcite samples, scanning electron microscopy (SEM) was performed using a S-4800 microscope (Hitachi) operated at an accelerating voltage of 10 kV. Samples were spread homogenously over a silicon wafer and measured at room temperature. The amount of impurities was determined by ICP-MS (inductively coupled plasma mass spectrometry, ELAN9000 Perkin Elmer) after dissolution of the material in nitric acid.

### Reagents

The Eu^3+^ stock solution was prepared by dissolving EuCl_3_⋅6H_2_O (Sigma Aldrich p.a.) in Milli-Q water (18.2 MΩ cm^−1^). The concentration of this stock solution, 1 × 10^−4 ^mol L^−1^, was confirmed by ICP-MS. All experiments were then carried out using diluted fractions of this solution. 0.01 mol L^−1^ NaCl and KCl solutions were prepared from a Merck powder (p.a.).

### Calcite recrystallization rates

For the determination of recrystallization rates of calcite we applied a method previously shown by Berner *et al*.^32^. For this, the radioactive tracer ^45^Ca was used to measure the uptake of calcium ions and calculate the amount of calcite recrystallized. The formula developed by Berner *et al*.^32^ was also used for this study:


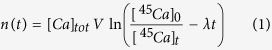


with n(t) as the time-dependent molar amount of recrystallized calcite, the total solution volume V, ^45^Ca concentrations at t = 0 and t, the decay constant λ = ln(2)/t_1/2_ of ^45^Ca radioactive decay and reaction time t. Two series of experiments with different solid-to-liquid ratios were conducted to determine recrystallization rates of the three calcite powders considered in this study. Firstly, a high ratio of 20 g L^−1^ was chosen for better comparability with Berner’s data. Due to the high recrystallization rates of calcites C2 and C3, equilibrium of the exchange reaction was reached after several hours and no rate determination could be conducted. Therefore, we performed a second experimental series with a lower solid-to-liquid ratio of 2.5 g L^−1^. The suspensions for both series were pre-equilibrated for one day and spiked with 15.5 MBq ^45^Ca as CaCl_2_ in 600 μL deionized water and shaken constantly in an overhead shaker. Sampling was performed within the first five days by removing 50 μL of solution and measuring the activity of ^45^Ca by liquid scintillation counting (Wallac WinSpectral, Ultima Gold LSC Cocktail, Perkin Elmer). The rates were determined in the linear regime by fitting the slope of the amount of recrystallized calcite as a function of time. Since the exchange reaction exhibits two time-dependent phases with distinct rates, only the first rate was determined in this study, being more sensitive to experimental conditions.

### Sorption experiments

Prior to the sorption experiments, all three calcites were equilibrated in 0.01 mol L^−1^ NaCl under atmospheric CO_2_ until a pH of 8.3 ± 0.1 was reached. Afterwards, supernatants were collected by filtration or centrifugation at 6,800 g for 1.5 h (Avanti J-20 XP Beckman Coulter). 125 mg of each fresh calcite were suspended in 50 mL of these pre-equilibrated supernatants (in polypropylene tubes) to obtain a final solid-to-solution ratio of 2.5 g L^−1^. Required amounts of the Eu^3+^ stock solution were added to reach a final concentration of 10^−6^ or 5 × 10^−7 ^mol L^−1^. The pH of the calcite suspensions was regularly checked and was found to be constant at 8.4 ± 0.1. All pH measurements (pH-meter Inolab WTW series pH720) were performed using a combination glass electrode (BlueLine 16 pH from Schott Instruments) in which an Ag/AgCl reference electrode was incorporated. The pH values were measured to an accuracy of ±0.05. Electrodes were calibrated using two NIST-traceable buffer solutions (pH 6.87 and pH 9.18 from WTW).

All experiments were performed at room temperature with a contact time of up to 3 years. After varying intervals, samples were centrifuged during 2 hours at 6,800 g and the remaining europium concentration in the supernatant was determined by ICP-MS. The difference to the initial europium content provided the amount of sorbed Eu^3+^. The calcite samples were dried at room temperature and subsequently analyzed by site-selective TRLFS. Their content in Ca and Eu at the end of the sorption experiments was determined by ICP-MS after dissolution. For the calcite C3, additional similar experiments were performed using calcite saturated solutions in 0.01 mol L^−1^ KCl.

### Site-selective TRLFS

The solid samples collected at the end of the sorption experiments were cooled down in a cryostat chamber at ultra-high vacuum (10^−6^–10^−7^ mbar). Three laser systems were used for these studies. The description of two systems can be found elsewhere^29^. For the third one, a Nd-YAG (Continuum SL I-20, Continuum, San Jose, USA) pumped dye laser (NarrowScan K, Radiant Dyes Laser & Accessories GmbH, Wermelskirchen, Germany) with Pyrromethene 580 (Radiant Dyes) as lasing dye was used.

Eu speciation was determined by adjusting the laser’s emission wavelength in steps of down to 0.01 nm through the spectral range of the non-degenerate (^5^D_0_ → ^7^F_0_) transition (575–582 nm). For each step the integrated fluorescence intensity is recorded. As the transition is non degenerate (J = 0 for both states), the number of peaks in such an excitation spectrum corresponds directly to the number of Eu^3+^ species in the system. Subsequently, these species can be excited directly to obtain single species, emission spectra and fluorescence lifetimes so far as spectral overlap permits. All site-selective TRLFS measurements were carried out at low temperatures (<10 K), in order to increase the spectral resolution. A spectrograph (Shamrock 303i, Andor Technology Ltd., Belfast, UK) with polychromator grids (300, 600 and 1200 lines per mm) and intensified CCD detector (ANDOR iStar 734 cooled to −20 °C to reduce thermal noise effects) were used to record the luminescence emission. For lifetime measurements, the camera delay was increased gradually up to several milliseconds, until fluorescence intensity dropped to a minimum of 1/e.

## Additional Information

**How to cite this article**: Hellebrandt, S.E. *et al*. Incorporation of Eu(III) into Calcite under Recrystallization conditions. *Sci. Rep.* **6**, 33137; doi: 10.1038/srep33137 (2016).

## Supplementary Material

Supplementary Information

## Figures and Tables

**Table 1:** Main characteristics of the different calcite materials C1, C2, and C3.

Calcite	Specific surface area (m^2^ g^−1^)	Recrystallization rate (μmol d^−1^)	Total concentration impurities (ppm)
C1	0.6 ± 0.1	—	38
C2	1.1 ± 0.1	8.9 ± 0.3	337
C3	18.0 ± 0.1	32.4 ± 5.7	2372

**Table 2:** Characteristics of the observed species.

	α	β	γ	δ
λ_exc._ [nm]	578.2	578.4	578.8	579.2–579.5
t [μs]	3700 ± 350	2450 ± 150	3750 ± 450	700 ± 50–2300 ± 150
n(H_2_O)	0.0	0.0	0.0	0.9–0.0 ± 0.5
symmetry	Low	Low	Low	Low
