# Supplementary material for: Incorporation of Eu(III) into Calcite under Recrystallization conditions
Source: Sci Rep. 2016 Sep 13;6:33137. doi: 10.1038/srep33137 (PMC5020613; doi:10.1038/srep33137)
Supplement: Supplementary Information [file srep33137-s1.pdf]

Supplementary information

Incorporation of Eu(III) into Calcite under Recrystallization conditions

S.E. Hellebrandt[1], S. Hofmann[1], N. Jordan[1], A. Barkleit[1], M. Schmidt[1,*]

[1]Helmholtz-Zentrum Dresden - Rossendorf, Institute of Resource Ecology, Bautzner Landstraße 400, 01328 Dresden, Germany

**Corresponding author:*

Phone: ++49 351 260 3136, e-mail: moritz.schmidt@hzdr.de

The Supplementary Information has 6 pages and contains 2 figures and 4 tables.

CALCITE CHARACTERIZATION

XRD

The three calcites were analyzed at room temperature on a Rigaku MiniFlex 600 diffractometer using Cu Kα radiation (λ=1.5406A°), operating in diffraction mode at 40 kV and 15 mA. The instrument was equipped with the one-dimensional semiconductor detector D/teX Ultra. Samples were measured in the 2θ range from 20 to 68° with steps of 0.01° (continuous scan with 5°/min). Samples were loaded onto Si zero-background sample holder and compressed lightly by a glass plate in order to obtain a smooth surface. The XRD pattern was compared with ICDD (International Centre of Diffraction Data) data for a qualitative characterization, using the PDXL Software (Rigaku). The results are presented in the Figure S1.

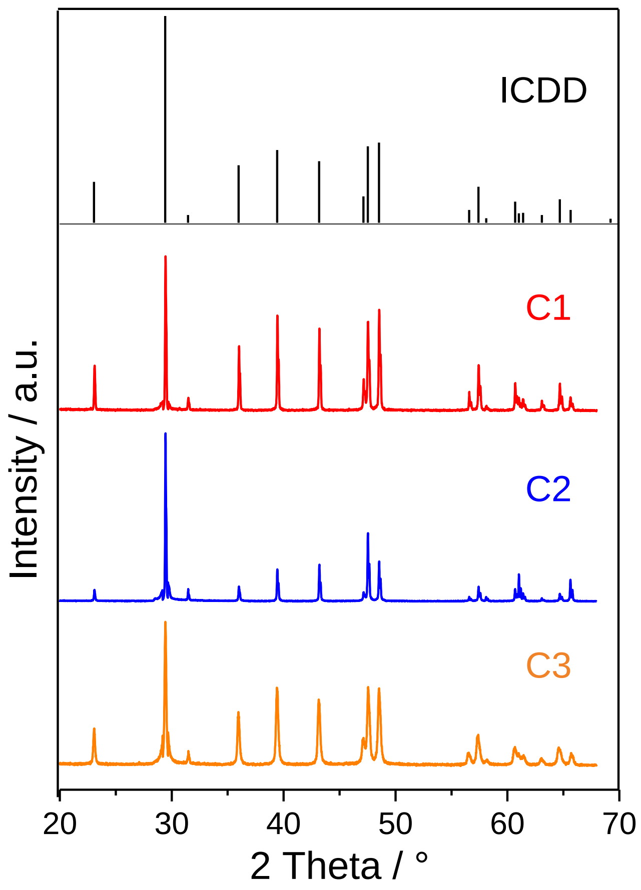

Figure S1: X-ray powder diffraction pattern of C1, C2 and C3 samples with reference pattern of calcite (ICDD card 01-083-0577) (for readability purposes, the intensity of the (104) diffraction line (29.4 °) was decreased).

As can be seen, the three materials correspond to a calcite phase, since the observed diffraction pattern matched well with the ICDD 01-083-0577 reference card. In addition, the

presence of other impurities or allotropic form of calcite, that is aragonite (ICDD card 01-071-2392) and vaterite (ICDD card 01-074-1867) were not observed.

Impurities

Table S1: Impurities [μg g^{-1}] of calcite C1, C2 and C3

**Probe**	**Calcite C1**	**Calcite C2**	**Calcite C3**
**Na**	<1	10.4	109
**Mg**	8.32	142	1390
**Si**	<10	37.1	389
**K**	<1	39.4	45.4
**Rb**	<0.01	<0.1	<0.1
**Sr**	14.5	52.6	156
**Cs**	<0.01	11.4	18.5
**Ba**	1.23	0.128	16.4
**Eu**	<0.01	0.039	0.027
**Fe**	7.48	21.2	192
**Mn**	<0.01	0.826	49.0
**Ni**	<0.01	0.523	1.17
**Zn**	<1	21.6	5.63

Fluorescence decay

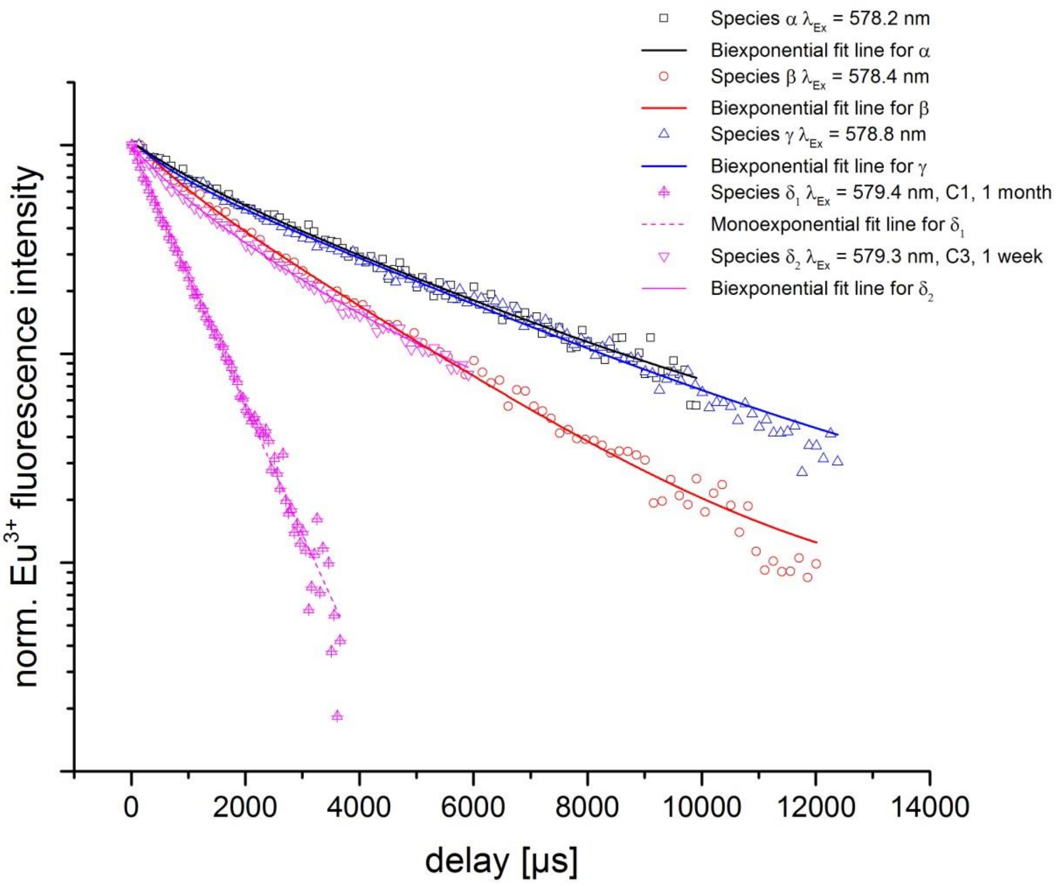

Figure S2: Fluorescence decay profiles of Eu(III) species α – δ after direct excitation of the respective $^7F_0 \rightarrow {}^5D_0$ transition at low temperatures (< 10 K)

Release of Na

Table S2: Release of Na from calcite C3 suspended in water (125 mg in 50 mL) after different reaction times, results of ICP-MS measurements

Reaction time [d]	Na [mol L^{-1}]
1	$8.1 \pm 0.8 \times 10^{-6}$
2	$7.5 \pm 0.8 \times 10^{-6}$
3	$7.2 \pm 0.7 \times 10^{-6}$
4	$8.2 \pm 0.8 \times 10^{-6}$
7	$8.4 \pm 0.8 \times 10^{-6}$
8	$8.5 \pm 0.9 \times 10^{-6}$
9	$7.8 \pm 0.8 \times 10^{-6}$
10	$9.0 \pm 0.9 \times 10^{-6}$

If all Na would be released from 125 mg calcite C3 in 50 mL water, it would reach a concentration of 1.18×10^{-5} mol L^{-1}. So nearly all Na goes into solution even after one day of reaction time, and after that, no more changes are observed.

Adsorption of Eu

Table S3: Adsorption of Eu onto calcite C1, C2 and C3 after different reaction times, results of ICP-MS measurements

	reaction time [d]	Eu removed from solution [%]
C1	30	82.0 ± 4.1
C2	9	98.2 ± 4.9
C2	30	99.8 ± 5.0
C2	60	97.0 ± 4.8
C2	380	99.8 ± 5.0
C3	7	99.9 ± 5.0
C3	30	99.9 ± 5.0
C3	60	99.9 ± 5.0
C3 KCl	30	100.0 ± 5.0

Table S4: Content of Eu in dependence of Ca in calcite after reaction, results of ICP-MS measurements

	**Solid-liquid ratio [g L^{-1}]**	**[Eu^{3+}] in reaction solution [mol L^{-1}]**	**Reaction time [d]**	**Eu in calcite [ppm]**
C1	1.5	2×10^{-6}	450	598 ± 60
C2	1.5	5×10^{-7}	380	89 ± 9
C3	2.5	1×10^{-6}	7	153 ± 15
C3	2.5	1×10^{-6}	30	149 ± 15
C3	2.5	1×10^{-6}	60	152 ± 15
C3 KCl	2.5	1×10^{-6}	30	161 ± 16
